# Nasal Obstruction Is Reduced 5–7 Years After Radiofrequency Turbinate Reduction: Results From a Retrospective Observational Cohort Study

**DOI:** 10.1111/coa.70148

**Published:** 2026-07-24

**Authors:** Veronica Ahlund, Pål Rosholm Comstedt, Maria Värendh, Pernilla Sahlstrand‐Johnson

**Affiliations:** ^1^ School of Medical Sciences, Faculty of Medicine and Health Örebro University Örebro Sweden; ^2^ Department of Otorhinolaryngology Örebro University Hospital Örebro Sweden; ^3^ Department of Clinical Sciences Lund University, Skåne University Hospital Lund Sweden; ^4^ Department of Otorhinolaryngology, Head and Neck Surgery Lund University, Skåne University Hospital Lund Sweden

**Keywords:** nasal obstruction, quality of life, radiofrequency therapy of the inferior turbinate, sleep quality, SNOT‐22

## Abstract

**Introduction:**

Nasal obstruction, often caused by structural and inflammatory conditions, is prevalent and significantly impacts health‐related quality of life. Radiofrequency Turbinate Reduction (RFTR) is a widely employed treatment for inferior turbinate hypertrophy, yet evidence of its long‐term effectiveness remains limited. This study aimed to assess the patient‐perceived long‐term outcomes of RFTR in patients with nasal obstruction, focusing on quality of life as measured by the Sinonasal Outcome Test‐22 (SNOT‐22) and to identify baseline factors predicting surgical outcome.

**Methods:**

A cohort of 198 patients aged ≥ 18 years who underwent RFTR for nasal obstruction filled out the SNOT‐22 questionnaire and additional questions preoperatively and 5–7 years postoperatively. Predictors of persistent nasal obstruction after RFTR were identified using logistic regression analysis.

**Results:**

SNOT‐22 scores significantly decreased from score mean 43.14 (0–104, min–max) to 34.66 (0–101) at the 5‐ to 7‐year follow‐up (*p* < 0.001), with notable improvements in the rhinologic and sleep function domains. However, baseline symptoms of ‘thick nasal discharge’ and ‘poor sleep quality’ were associated with a higher relative risk and higher odds ratio of continued nasal obstruction after adjustment for confounders. These baseline symptoms remained significant predictors of persistent nasal obstruction postoperatively.

**Conclusion:**

Radiofrequency Turbinate Reduction reduced nasal obstruction and also showed long‐term efficacy in improving health‐related quality of life after long‐term outcome, with most patients reporting positive outcomes. Patients with severe baseline symptoms of thick nasal discharge and poor sleep quality may experience less favourable outcomes, possibly due to comorbidities. These findings highlight the potential of SNOT‐22 to identify patients at risk of persistent symptoms post‐RFTR.

## Practitioner Points

In clinical practice, it is important for practitioners to recognise that Radiofrequency Turbinate Reduction is likely to provide long‐term improvement in nasal obstruction. However, patients with thick nasal discharge or significant sleep disturbances have been shown to have a higher risk of persistent severe nasal obstruction.

## 1 Introduction

Nasal obstruction is a common symptom among patients referred to otorhinolaryngologists, with a variety of underlying causes, both anatomical and structural. Causes include septal deviation, nasal polyps, inferior turbinate hypertrophy, chronic rhinosinusitis and both allergic and non‐allergic rhinitis [1]. Nasal obstruction can significantly impact health‐related quality of life (HRQOL), leading to additional symptoms such as loss of smell and taste and impaired sleep quality [2].

Hypertrophy of the inferior turbinate is usually first treated with conservative methods such as intranasal steroids, antihistamines, sympathomimetics and, if unsuccessful, surgical turbinate interventions [3].

Radiofrequency Turbinate Reduction (RFTR) is one of the most common techniques for turbinate reduction [2] and is widely regarded as a safe and effective procedure [4, 5, 6, 7, 8]. The surgery involves submucosal tissue heating, leading to tissue fibrosis during the healing process, which results in a sustained reduction in turbinate volume over time [9].

Previous research has focused mainly on the short‐term outcomes of RFTR for isolated inferior turbinate hypertrophy [10, 11]. Long‐term studies are limited, typically covering a few years with small sample sizes [12, 13]. These studies, using both clinical assessments and patient‐reported outcomes, consistently show that RFTR leads to short‐term improvements in turbinate hypertrophy and nasal obstruction compared to controls [10, 11]. Positive effects from surgery have been reported to last up to 3 years [12, 13].

Various patient‐reported outcome measures have been used to evaluate the effectiveness of RFTR. The 22‐item Sino‐Nasal Outcome Test (SNOT‐22) is a validated questionnaire originally developed to assess the impact of chronic rhinosinusitis (CRS) [14] and has also been utilised to evaluate septoplasty outcomes [15]. Although SNOT‐22 has not yet been validated for use following RFTR, it may still offer clinically relevant insights as a quality‐of‐life assessment tool, particularly in view of the currently limited number of validated alternatives available for this purpose.

The primary aim of the present study was to investigate the long‐term effectiveness of RFTR in a larger cohort of patients with nasal obstruction, using SNOT‐22 to measure both nasal blockage and quality of life. A secondary aim was to identify factors from the baseline scoring of SNOT‐22 that could predict a lack of improvement in nasal obstruction after RFTR. The study hypothesised that the positive effects observed postoperatively would not be sustained over an extended period.

## 2 Material and Methods

### 2.1 Study Population and Design

Patients aged ≥ 18 years with nasal obstruction who had not responded adequately to nasal steroids after a recommended treatment period of 2 months and in whom clinical examination demonstrated hypertrophy of the inferior turbinates were referred for RFTR at Skane University Hospital, between 8 March 2011 and 30 June 2013. Patients with other conditions causing nasal obstruction, such as nasal septum deviation or internal/external nasal valve collapse or stenosis, were excluded from the current study and referred for surgery addressing those specific issues. These RFTR patients were all offered the opportunity to fill out the SNOT‐22 questionnaire prior to surgery. In total, 290 patients completed the questionnaires prior to RFTR. They were subsequently contacted by mail and invited to participate in a long‐term follow‐up 5–7 years post‐surgery. Reminders were sent by mail and patients were also called if there was no response. Along with the SNOT‐22, the patients received an additional questionnaire addressing health‐related conditions that included questions about their experience of the RFTR treatment, allergies, body measurements and comorbidities. The SNOT‐22 and the accompanying questionnaire, along with written informed consent, were returned to the study coordinator and data were recorded anonymously in a database. In 2018, the Regional Ethics Review Board in Lund (registration number 2018/976) approved the study protocol.

### 2.2 Radiofrequency Turbinate Reduction (RFTR)

All patients were treated with the same procedure by one of three surgeons. The inferior turbinate was topically anaesthetised using local anaesthesia (Carbocain without adrenaline) in an outpatient facility. The radio frequency energy was delivered with a handheld bipolar probe with two needles, set at bipolar coagulation mode, inserted in the mucosa along the full length of the inferior turbinate, producing a temperature of 60°C–90°C. The radiofrequency generator was a Sutter, BM‐780 II Radiofrequency Unit, D‐79108 Freiburg, Germany. The procedure is known to be well tolerated under local anaesthesia [16].

### 2.3 Sinonasal Outcome Test‐22

The Sinonasal Outcome Test‐22 (SNOT‐22) is a health‐related quality of life (HRQOL) questionnaire derived from the SNOT‐20, originally developed by Piccirillo et al. in 2002 [17]. Two additional questions were incorporated to address nasal blockage and the loss of sense of taste and smell [17]. Each of the 22 criteria is scored on a scale from 0 to 5, yielding a total score range from 0 to 110, where lower scores indicate a better HRQOL. Although SNOT‐22 was initially designed for use in patients with chronic rhinosinusitis (CRS), it has become a widely used instrument for evaluating outcomes in various procedures [14, 18, 19]. The questionnaire has been translated and validated in multiple languages, including Swedish [20].

To facilitate clinical interpretation and focus on disease‐specific symptoms, SNOT‐22 is divided into four domains: rhinology (items 1, 2, 3, 4, 5, 7 and 8), ear/facial symptoms (items 6, 9, 10, 11 and 12), sleep function (items 13, 14, 15 and 16) and psychological issues (items 17, 18, 19, 20, 21 and 22) [21].

### 2.4 Characteristics of the Patients and Medical History

A health‐related questionnaire was also distributed at the 5–7‐year follow‐up. The questionnaire included 11 relevant questions with response options categorised as affirmative, negative and unknown.

### 2.5 Statistical Analysis

The results from all items on the SNOT‐22 questionnaire were presented as mean values. The Wilcoxon signed‐rank test was used to assess the significance of differences between the mean SNOT‐22 scores obtained preoperatively and at the five‐ to seven‐year postoperative follow‐up. In addition, the median total SNOT‐22 score was calculated together with interquartile range (IQR) for both the preoperative assessment and the five‐ to seven‐year postoperative follow‐up.

Subsequently, responses were categorised based on the percentage of patients reporting severe symptoms. Responses were dichotomised into two categories: severe and mild. A severe condition was defined as a score of 4–5 on the relevant SNOT‐22 item, whereas a mild condition was defined as a score of 0–3. The Chi‐square test was applied to evaluate the significance of differences between these categories—severe and mild—when comparing preoperative and postoperative SNOT‐22 scores.

The SNOT‐22 item on severe nasal blockage at the 5–7 year follow‐up was dichotomised into severe and mild blockage to identify preoperative predictors of outcome. Preoperative SNOT‐22 responses were compared between groups, categorising each criterion as severe versus non‐severe. Differences were expressed as relative risk.

The McNemar test was used to compare severe versus mild nasal obstruction at the 5–7 year follow‐up with the corresponding severe versus mild conditions for all SNOT‐22 questions in the preoperative setting.

Additionally, an odds ratio was calculated for the 10 items that demonstrated the highest relative risk. Multiple regression analysis was subsequently performed to evaluate factors that could predict persistent nasal obstruction 5–7 years after surgery.

Simple mean imputation was used for missing data in SNOT‐22 [22]. The mean value of the available responses was calculated and used to fill in the missing items, provided that at least 50% of the questions had been answered.

A two sided *p*‐value ≤ 0.05 was considered significant. All analyses were conducted using SPSS version 29.0.0.0.

## 3 Results

A total of 295 patients aged ≥ 18 completed the preoperative SNOT‐22. Five were excluded due to incomplete responses (< 50% answered). The study flow is shown in Figure 1. At the 5‐year follow‐up, 92 patients did not respond, leaving 198 patients (81 female) with a mean age of 43 years (min–max 18–77). Simple mean imputation was applied to 45 preoperative cases (16%) and 15 follow‐up cases (8%) with missing data. On average, fewer than two items were missing per incomplete questionnaire at both time points.

**FIGURE 1:**
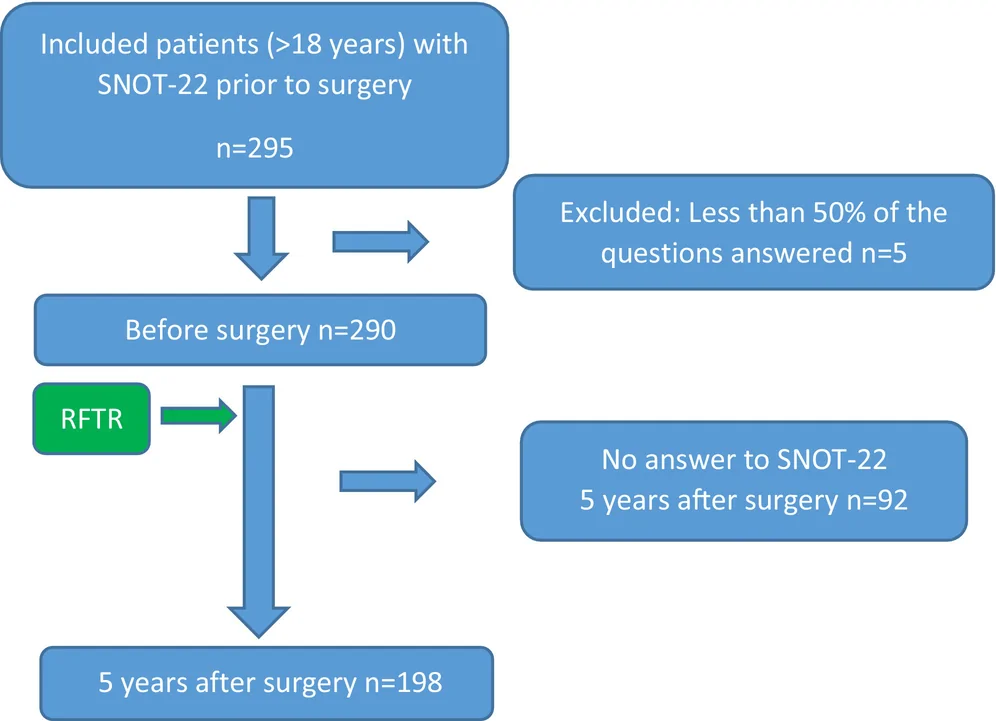
Flowchart of the study inclusion of patients with the age ≥ 18 years. RFTR, radiofrequency turbinate reduction, surgery; SNOT‐22, Sinonasal Outcome Test.

The response rate after 5–7 years was 68% (*n* = 198). There were no sex differences between responders and non‐responders (*p* = 0.45), nor in mean SNOT‐22 scores (*p* = 0.08). However, non‐responders were significantly younger than responders (33 vs. 43 years, *p* < 0.001).

Although only 69 patients (35%) were satisfied with the surgery, according to the patient medical characteristics questionnaire, 129 (65%) would undergo it again (Table 1). Analysis of the dichotomised groups showed that 29% of those with mild nasal obstruction were satisfied, compared to 6% of those with severe obstruction.

**TABLE 1:** Patient characteristics and medical history.

*n* = 198			
	Affirmative	Negative	Unknown
Satisfied with RFTR	35%	47%	18%
Would still do it with current knowledge	65%	32%	3%
Other RFTR	24%	76%	0%
Other nasal surgery post RFTR	10%	90%	0%
Allergy	32%	48%	20%
Snore	61%	23%	16%
Sleep apnea	19%	77%	4%
Hypertension	23%	75%	2%
Pulmonary condition	16%	77%	8%
Heart condition	9%	88%	4%

	Affirmative	Negative	Previous
Smoker	8%	72%	20%

	Mean	Min	Max
Height (m)	1.75	1.56	1.95
Weight (kg)	80.2	42	145
BMI	26.08	15.24	44.53

*Note:* Patient characteristics and medical history as patient reported in questionnaire regarding health‐related conditions after 5–7 years.

Abbreviations: BMI, body mass index; *n*, number of patients; RFTR, radiofrequency turbinate reduction.

The total SNOT‐22 score significantly decreased from a mean of 43.14 (range 0–104) preoperatively to 34.66 (0–101) 5–7 years postoperatively (*p* < 0.001). In terms of the median, the total SNOT‐22 score decreased from 41 to 31, with an interquartile range (IQR) of 27 preoperatively and 34 5–7 years postoperatively. There was an improvement in the criterion related to nasal blockage/congestion. All individual SNOT‐22 item scores, except for four ‘runny nose’, ‘dizziness’, ‘facial pain/pressure’ and ‘sad’, decreased significantly (*p* < 0.05) (Table 2).

**TABLE 2:** SNOT‐22 mean score.

		Preoperative score	Postoperative score 5 years	Change	*p*
1.	Need to blow nose	2.86	2.43	−0.43	**< 0.001**
2.	Sneezing	1.89	1.62	−0.28	**0.004**
3.	Runny nose	2.07	1.93	−0.14	0.161
4.	Blockage/congestion of nose	3.82	2.84	−0.98	**< 0.001**
5.	Sense of taste/smell	1.79	1.39	−0.40	**< 0.001**
6.	Cough	1.37	1.12	−0.25	**0.013**
7.	Post nasal discharge	2.27	1.84	−0.42	**< 0.001**
8.	Thick nasal discharge	1.69	1.19	−0.49	**< 0.001**
9.	Ear fullness	1.56	1.20	−0.36	**0.003**
10.	Dizziness	0.81	0.76	−0.05	0.587
11.	Ear pain	0.72	0.47	−0.25	**0.007**
12.	Facial pain/pressure	0.89	0.79	−0.10	0.265
13.	Difficulty falling asleep	1.88	1.36	−0.52	**< 0.001**
14.	Waking up at night	2.54	2.19	−0.35	**0.007**
15.	Lack of a good night's sleep	2.46	2.05	−0.41	**0.001**
16.	Waking up tired	2.97	2.37	−0.60	**< 0.001**
17.	Fatigue	2.55	2.10	−0.44	**< 0.001**
18.	Reduced productivity	1.9	1.56	−0.34	**0.003**
19.	Reduced concentration	1.78	1.45	−0.33	**0.004**
20.	Frustrated/restless/irritable	1.86	1.31	−0.55	**< 0.001**
21.	Sad	1.01	1.03	0.02	0.942
22.	Embarrassed	2.45	1.66	−0.79	**< 0.001**
	Total	43.14	34.66	−8.48	**< 0.001**

*Note:* The actual scores of SNOT‐22 depicted prior to surgery and 5–7 years after RFTR, showing improvement in nasal obstruction and in all items except for four, 5–7 years after surgery. *n* = 198 patients. Bold values indicate statistical significance (*p* < 0.05).

Analysis of the dichotomised data, comparing the percentage of patients rating > 3 (severe condition) versus 0–3 (mild condition) for each criterion, revealed significant changes (*p* < 0.05) at the 5–7 year follow‐up. The rhinological domain showed changes 5–7 years post‐surgery (Figure 2). Additionally, the sleep function domain demonstrated significant improvements, with a shift from severe to mild conditions 5–7 years after RFTR.

**FIGURE 2:**
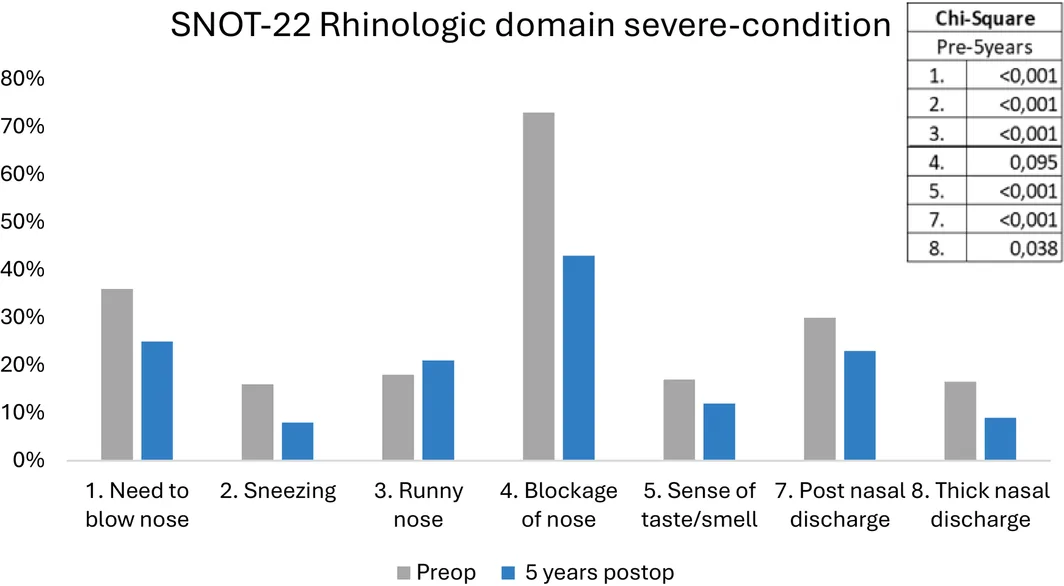
Patients who scored severe conditions in the rhinologic domain of SNOT‐22 showed significant differences (< 0.05) in all criteria, apart from blockage of nose, in the scores before RFTR compared to 5–7 years later. Due to the dichotomisation into the two groups, severe and mild condition and the fact that chi square compares four groups, which in this case were too similar, we could not show any statistical significance in blockage of nose, although a clear difference was observed.

Analysis of the five‐ to seven‐year outcome of RFTR for nasal blockage, the primary indication for surgery, showed that 113 out of 198 patients rated their nasal obstruction as 1–3 (mild). The remaining 85 patients rated their obstruction as 4–5 (severe). Preoperatively, there were 54 patients with mild nasal obstruction and 144 patients with severe nasal obstruction. We have performed a sensitivity analysis excluding the 10% who had undergone additional nasal surgery and the mean SNOT‐22 result did not change.

The relative risk of severe nasal obstruction at the five‐ to seven‐year follow‐up was assessed by comparing all baseline SNOT‐22 scores for severe conditions between patient groups experiencing mild and severe nasal obstruction 5–7 years post‐RFTR. A relative risk greater than 1.5 was considered indicative of an increased likelihood of persistent severe nasal obstruction. Ten SNOT‐22 criteria showed a significantly higher relative risk (RR > 1.5) for severe nasal obstruction after 5–7 years, when baseline scores were for severe conditions (Table 3).

**TABLE 3:** Relative risk severe condition.

Severe condition (score 4–5) for the SNOT‐22 items at baseline	Severe nasal obstruction at 5 years	Mild nasal obstruction at 5 years	McNemar	Relative risk
	*n* = 85	*n* = 113	*p*	
1. Need to blow nose	47%	28%	0.171	1.66
2. Sneezing	18%	15%	< 0.001	1.17
3. Runny nose	25%	13%	< 0.001	**1.86**
4. Blockage/congestion of nose	79%	68%	< 0.001	1.16
5. Sense of taste/smell	21%	13%	< 0.001	**1.60**
6. Cough	9%	11%	< 0.001	0.89
7. Post nasal discharge	34%	26%	0.005	1.33
8. Thick nasal discharge	24%	12%	< 0.001	**1.90**
9. Ear fullness	15%	16%	< 0.001	0.96
10. Dizziness	8%	6%	< 0.001	1.33
11. Ear pain	5%	4%	< 0.001	1.06
12. Facial pain/pressure	11%	6%	< 0.001	**1.71**
13. Difficulty falling asleep	28%	15%	< 0.001	**1.88**
14. Waking up at night	41%	30%	0.101	1.37
15. Lack of a good night's sleep	41%	25%	0.017	**1.66**
16. Waking up tired	52%	43%	0.461	1.19
17. Fatigue	34%	28%	0.014	1.20
18. Reduced productivity	25%	13%	< 0.001	**1.86**
19. Reduced concentration	16%	10%	< 0.001	**1.69**
20. Frustrated/restless/irritable	21%	12%	< 0.001	**1.84**
21. Sad	9%	3%	< 0.001	**3.55**
22. Embarrassed	35%	33%	< 0.001	1.08

*Note:* Comparison of the two groups for severe versus mild nasal obstruction after 5–7 years. The values represent severe condition (4–5 score) response rates at baseline for each item (*n* = 198). *p* < 0.05 is considered statistically significant. Bold values indicate statistically significant SNOT‐22 items with RR > 1.5.

To determine the odds of persistent severe nasal obstruction (scoring 4–5) at the five‐ to seven‐year follow‐up, odds ratios (OR) were calculated for these 10 SNOT‐22 criteria. Five of the 10 items showed statistically significant associations in the unadjusted analysis. After adjusting for confounders, including smoking, allergy and sex, two items, ‘Thick nasal discharge’ and ‘Lack of a good night's sleep’ remained significant (Table 4).

**TABLE 4:** Odds ratio severe condition.

	Unadjusted odds ratio (95% CI)	*p*‐value for group difference	Adjusted odds ratio (95% CI)	*p*‐value for group difference
3. Runny nose	2.14 (1.03–4.47)	**0.04**	2.33 (0.84–6.48)	0.1
5. Sense of taste/smell	1.76 (0.83–3.73)	0.14	1.48 (0.51–4.30)	0.47
8. Thick nasal discharge	2.18 (1.03–4.61)	**0.04**	3.02 (1.10–8.28)	**0.03**
12. Facial pain/pressure	1.79 (0.64–5.03)	0.27	1.18 (0.26–5.29)	0.83
13. Difficult falling asleep	2.22 (1.10–4.47)	**0.03**	1.36 (0.53–3.50)	0.52
15. Lack of a good night's sleep	2.13 (1.16–3.90)	**0.02**	2.49 (1.06–5.85)	**0.04**
18. Reduced productivity	2.25 (1.03–4.47)	**0.04**	1.99 (0.71–5.58)	0.19
19. Reduced concentration	1.83 (0.79–4.26)	0.16	1.46 (0.49–4.37)	0.5
20. Frustrated/restless/irritable	2.07 (0.95–4.50)	0.07	2.63 (0.86–8.03)	0.09
21. Sad	3.81 (0.98–14.82)	0.054	3.66 (0.63–21.30)	0.15

*Note:* Prediction of severe nasal obstruction after 5–7 years depending on severe SNOT‐22 criterion grading at baseline. OR calculated for the difference between the two groups, severe versus mild nasal obstruction after 5–7 years, for the 10 SNOT‐22 criteria with RR > 1.5. Calculations made for unadjusted as well as adjusted OR (*n* = 198 patients). Bold values indicate statistical significance (*p* < 0.05).

## 4 Discussion

In this retrospective cohort study, a significant improvement in nasal obstruction measured by HRQOL assessments through SNOT‐22 was still evident at 5–7 years post RFTR. Our primary aim was met and we demonstrated the long‐term effectiveness of RFTR treatment. We identified factors from the baseline scoring that could predict lack of improvement, which was our secondary aim. We hypothesised that the positive postoperative effects would diminish over time. However, we were surprised to find the improvements proved to be sustained in the long term.

In the current study, the patients had poor quality of life related to nasal health symptom (SNOT‐22 mean scores 43.14) preoperatively. Other nasal surgery studies involving patients with similarly elevated preoperative SNOT‐22 scores have reached comparable conclusions [18].

There was a statistically significant improvement in nasal obstruction and HRQOL measured by SNOT‐22 5–7 years after RFTR and this improvement was observed in approximately 80% of the SNOT‐22 criteria. Previous studies in patients with chronic rhinosinusitis have identified a minimal clinical difference of total SNOT‐22 score as mean 8.9 units [14, 23]. In this study, involving patients treated with RFTR for nasal obstruction, the mean total difference in SNOT score was 8.48 units and the median total difference 10 units, respectively. In relation to other studies, this change can be considered to be clinically meaningful. The key symptom in this population was nasal blockage and there was indeed a decrease by one unit in the item ‘Blockage/congestion of nose’ of SNOT‐22.

Several previous studies have reported similar short‐term HRQOL improvements up to 1 year post‐ RFTR [3, 6], as well as long‐term benefits extending up to three years [9, 12, 13]. These studies utilised VAS or SNOT‐22 as assessment, with sample sizes ranging from 19 to 92 patients. However, there is a notable scarcity of studies with larger sample sizes and longer follow‐up periods. No other studies reporting a five‐ to seven‐year follow‐up post‐ RFTR were identified in the literature.

Data dichotomised based on degree of improvement have been utilised to evaluate RFTR outcomes. One such study reported HRQOL improvements similar to our findings but with a follow‐up limited to 1 year [24].

Previous research has investigated factors that influence the outcome of RFTR. Predictors such as anterior septal deviation, allergies and asthma have been associated with less favourable long‐term outcomes, whereas older age and non‐smoking status have been linked to improved long‐term outcomes [24, 25]. These studies primarily focused on patient characteristics and medical history as predictors. In contrast, our study analysed HRQOL conditions at baseline, using the SNOT‐22 questionnaire as a potential predictive tool for RFTR outcomes. Thus, RFTR appears to result in subjective long‐term improvements in nasal obstruction and quality of life.

When investigating factors influencing remaining nasal symptoms our analysis identified severe symptoms in 10 items that demonstrated a significant relative risk (> 1.5) of refractory severe nasal obstruction. A high score in the questions ‘Thick nasal discharge’ and ‘Lack of a good night's sleep’ preoperatively was predictive of a higher odds of insufficient response to RFTR for nasal obstruction after surgery. It can be hypothesised that patients scoring high on the item ‘Thick nasal discharge’ may suffer from chronic rhinosinusitis, for which RFTR may not be an appropriate treatment option, alternatively extended treatment planning. Similarly, ‘Lack of a good night's sleep’ could reflect underlying sleep disturbances, such as obstructive sleep apnoea or insomnia. These patients may benefit from managing their sleep disturbances with targeted treatment before assessing nasal obstruction and selecting the most appropriate treatment approach.

Although two thirds of the cohort stated they would undergo the procedure again with their current knowledge, only a bit over one third reported actually being satisfied with the RFTR treatment. This suggests that while the surgery may provide measurable, meaningful symptom relief on the SNOT‐22, it routinely fails to meet the subjective definitions of a successful or complete outcome for the patients. The satisfaction of the patients might depend on factors beyond symptom reduction alone, such as residual complaints, comorbid conditions, or unmet expectations. Or is it possible that the patients believe that another surgery might give even more symptom relief? The fact that a majority of patients would nevertheless choose to undergo the procedure again indicates that the perceived benefit was meaningful, despite insufficiency to meet the subjective definition of treatment success for the patients. These findings highlight the importance of thorough preoperative patient counselling, with an emphasis on setting realistic expectations and clarifying that partial rather than complete symptom resolution is the most likely outcome.

Our study has demonstrated the long‐term positive effect of RFTR on nasal obstruction. Additionally, we investigated the potential of utilising HRQOL questionnaires to identify predictors of treatment failure to reduce nasal blockage through RFTR. Among the SNOT‐22 criteria, scoring high in ‘Thick nasal discharge’ and ‘Lack of a good night's sleep’ were identified as significant risk factors. However, further research is needed to establish a comprehensive and reliable list of other predictive factors.

A major strength of this study is the use of a validated and relevant questionnaire, providing detailed insights into nasal symptoms and related issues, such as sleep quality. Additionally, while the relatively large sample size allows for a robust analysis of symptom prevalence and patterns within the studied cohort, one should be careful when considering generalisation to the general population. Important factors such as race, ethnicity and age groups were not specifically accounted for, which limits the applicability of the results across more diverse populations. Unlike a strictly selected research cohort, this study reflects real‐world clinical settings, which can be viewed as both a strength and a limitation.

One primary limitation of the study is the reliance on subjective, patient‐reported outcomes, which lack the objectivity of physiological measures. While the SNOT‐22 captures patient‐perceived symptom burden and health‐related quality of life, it does not provide objective information on nasal airflow or anatomical changes. The absence of objective assessments, such as rhinomanometry or peak nasal inspiratory flow, limits the ability to correlate subjective improvement with measurable changes in nasal patency. However, the results are derived from a validated tool, giving important patient perspectives. Future studies should pair patient‐reported outcome measures with objective physiological measures to better assess efficacy and clarify long‐term outcomes.

The absence of a control group is another important limitation. Although the inclusion of a non‐treated control group would have strengthened causal inference and improved the reliability of the findings, ethical considerations complicate such study designs, as patients were selected for surgery due to insufficient response to medical therapy. Nevertheless, without a control group, it cannot be definitively proven that the sustained improvement over 5–7 years is caused by the RFTR. Natural disease fluctuations, regression to the mean, or the effects of concomitant medical treatments cannot be ruled out in this study but can be addressed in future ones.

A further limitation of this study is the lack of detailed diagnostic information, including comorbid conditions and the duration of prior medical treatment. Factors like one fifth reporting sleep apnoea and one third reporting allergies might influence rhinologic and sleep domains of the SNOT‐22 and treatment outcomes. However, the cohort reflects typical clinical practice, where patients selected for RFTR generally present with nasal obstruction due to a combination of turbinate hypertrophy and rhinitis, making the study population representative of those who routinely undergo this procedure.

A further limitation of this study is the lack of detailed diagnostic information, including comorbid conditions and the duration of prior medical treatment; factors such as roughly one fifth reporting sleep apnoea and one third reporting allergies may influence the rhinologic and sleep domains of the SNOT‐22 and, consequently, treatment outcomes. The cohort reflects typical clinical practice, where patients selected for RFTR generally present with nasal obstruction due to a combination of turbinate hypertrophy and rhinitis, making the study population representative of those who routinely undergo this procedure. Additionally, up to 25% had prior nasal surgery and 10% underwent further surgery after RFTR, complicating attribution of long‐term quality‐of‐life gains to RFTR alone. This nevertheless reflects routine practice with multiple treatments before RFTR. The findings suggest repeated RFTR may be preferable in cases of recurrence. A sensitivity analysis excluding the 10% showed no change in the mean SNOT‐22.

An additional constraint is the loss of one third to follow‐up and also the observed age difference between responders and non‐responders. Although the overall response rate was acceptable for a long‐term follow‐up study, non‐responders were significantly younger than responders. This age‐related response pattern introduces a potential risk of response bias, as older patients may differ from younger patients in symptom perception, treatment expectations and long‐term disease progression. Previous studies have shown that older patients are more likely to respond and also more likely to report lower scores than younger patients [26]. This may have contributed to an overestimation of the perceived long‐term efficacy of RFTR. Consequently, the results are less generalisable to younger populations, so future studies should minimise loss to follow‐up and ensure age‐balanced response rates.

Despite these limitations, with the validated QoL tool, the findings provide a meaningful foundation for future research and can support clinical decision making.

As part of the broader context of nasal surgery, it is relevant to note that RFTR is one frequently used rhinosurgical method. Other methods to be mentioned are total and partial turbinectomy which can improve obstruction but are associated with higher rates of crusting, bleeding and impaired turbinate function. More conservative methods such as laser, electrocautery and cryotherapy have variable complication profiles and limited effect. Turbinate out fracture alone has not shown reliable efficacy [27].

Among submucosal techniques, coblation is a gentle, low‐risk method based on a plasma‐mediated, low‐temperature process. When comparing to RFTR, both techniques are associated with minimal complications and limited postoperative discomfort and have been shown to provide improvements in airflow and symptoms [28]. Microdebrider‐assisted resection, on the other hand, may yield more durable outcomes but at the expense of greater invasiveness and higher risk of bleeding or synechiae [29]. In this context, RFTR remains a practical and widely used option, combining efficacy with low complication rates and technical simplicity. Importantly, as previous studies have not consistently demonstrated long‐term effectiveness, largely due to small cohorts and short follow‐up [28], the present study was undertaken to address this gap and demonstrates that RFTR treatment provides sustained long‐term benefit. These findings underline the importance of evaluating the long‐term outcomes of comparable techniques in future research.

## 5 Conclusion

Radiofrequency Turbinate Reduction reduced nasal obstruction and showed long‐term efficacy in improving health‐related quality of life 5–7 years postoperatively, in a large majority of the patients. Patients' baseline symptoms of thick nasal discharge and poor sleep quality may experience less favourable outcomes, possibly due to comorbidities. These findings highlight the potential of SNOT‐22 to identify patients at risk of persistent symptoms post‐RFTR when considering patients prior to surgery.

## Author Contributions

V.A., P.R.C., M.V. and P.S.‐J. planned the project. P.R.C. collected the data and obtained ethical approval. V.A. analysed the data and wrote the manuscript. M.V. and P.S.‐J. supervised the project and reviewed the manuscript.

## Funding

The authors have nothing to report.

## Ethics Statement

From an ethical point of view it is important to evaluate the outcome of the often performed procedure Radiofrequency Turbinate Reduction (RFTR) and thereby improve treatment for patients. All patients signed an informed consent and data were de‐identified in a database to assure anonymity. The Regional Ethics Review Board at Lund University (registration number 2018/976) has approved the study protocol.

## Consent

Patients have signed a written informed consent.

## Conflicts of Interest

The authors declare no conflicts of interest.

## Supporting information


**Supporting Information: S1** Patients scoring severe conditions in the sleep function domain, showing significant improvement for all criteria (*p* < 0.05).

## Data Availability

The data that support the findings of this study are available from the corresponding author upon reasonable request.

## References

[1] A. H. Nilsen , A. S. Helvik , W. M. Thorstensen , and V. Bugten , “A Comparison of Symptoms and Quality of Life Before and After Nasal Septoplasty and Radiofrequency Therapy of the Inferior Turbinate,” BMC Ear, Nose and Throat Disorders 18 (2018): 1, 10.1186/s12901-017-0050-z. PMC5787231 29422774

[2] A. Kankaanpää , T. Harju , and J. Numminen , “The Effect of Inferior Turbinate Surgery on Quality of Life: A Randomized, Placebo‐Controlled Study,” Ear, Nose & Throat Journal 100, no. 10Suppl (2021): 1107S–1112S, 10.1177/0145561320927944. 32453643

[3] L. J. J. Bäck , M. L. Hytönön , H. O. Malmberg , and J. S. Ylikoski , “Submucosal Bipolar Radiofrequency Thermal Ablation of Inferior Turbinates: A Long‐Term Follow‐Up With Subjective and Objective Assessment,” Laryngoscope 112, no. 10 (2002): 1806–1812, 10.1097/00005537-200210000-00019. 12368620

[4] E. P. Prokopakis , E. I. Koudounarakis , and G. A. Velegrakis , “Efficacy of Inferior Turbinoplasty With the Use of CO_2_ Laser, Radiofrequency, and Electrocautery,” American Journal of Rhinology & Allergy 28, no. 3 (2014): 269–272, 10.2500/1jra.2014.28.4044. 24980241

[5] L. J. J. Bäck , T. Liukko , S. T. Sinkkonen , J. Ylikoski , and A. A. Mäkitie , “Complication Rates of Radiofrequency Surgery in the Upper Airways: A Single Institution Experience,” Acta Oto‐Laryngologica 129, no. 12 (2009): 1469–1473, 10.3109/00016480802706719. 19922099

[6] C. J. Naese and G. A. Krempl , “Radiofrequency Treatment of Turbinate Hypertophy: A Randomized, Blinded, Placebo‐Controlled Clinical Trial,” Otolaryngology—Head and Neck Surgery 130, no. 3 (2004): 291–299, 10.1016/j.otohns.2003.11.003. 15054369

[7] M. L. Hytönen , L. J. J. Bäck , A. V. Malmivaara , and R. P. Roine , “Radiofrequency Thermal Ablation for Patients With Nasal Symptoms: A Systematic Review of Effectiveness and Complications,” European Archives of Otorhinolaryngology 266, no. 8 (2009): 1257–1266, 10.1007/s00406-009-0914-y. 19189114

[8] G. Harsten , “How We Do It: Radiofrequency‐Turbinectomy for Nasal Obstruction Symptoms,” Clinical Otolaryngology 30, no. 1 (2005): 64–66, 10.1111/j.1365-2273.2004.00941.x. 15748194

[9] N. Neiderman , I. Caspi , N. Eisenberg , et al., “Quality of Life After Frequency Ablation Turbinate Reduction (RFATR) Among Patients With Rhinitis Medicamentosa & Withdrawal From Decongestant Topical Spray Abuse,” American Journal of Otolaryngology 44, no. 4 (2023): 103842, 10.1016/j.amjoto.2023.103842. 36989755

[10] G. M. Bran , S. Hünnebeck , R. M. Herr , K. Hörmann , and A Stuck BA , “Bipolar Radiofrequency Volumetric Tissue Reduction of the Inferior Turbinates: Evaluation of Short‐Term Efficacy in a Prospective, Randomized, Single‐Blinded, Placebo‐Controlled Crossover Trial,” European Archives of Otorhinolaryngology 270, no. 2 (2013): 595–601, 10.1007/s00405-012-2088-2. 22740153

[11] L. Di Reinzo Businco , A.,. L. Di Rienzo Businco , S. Laurino , and M. Lauriello , “Turbinoplasty With Quantic Molecular Resonance in the Treatment of Persistent Moderate‐Severe Allergic Rhinitis: Comparative Analysis of Efficacy,” American Journal of Rhinology & Allergy 28, no. 2 (2014): 164–168, 10.2500/1jra.2014.28.3990. 24717954

[12] T. Hariju and J. Numminen , “The Long‐Term Effect of the Inferior Turbinate Surgery Techniques on Nasal Obstruction and Quality of Life,” Annals of Otology, Rhinology, and Laryngology 131, no. 9 (2022): 933–940, 10.1177/00034894211049573. PMC9340137 34612723

[13] M. W. Porter , N. W. Hales , C. J. Nease , and G. A. Krempl , “Long‐Term Results of Inferior Turbinate Hypertrophy With Radiofrequency Treatment: A New Standard of Care?,” Laryngoscope 116, no. 4 (2006): 554–557, 10.1097/01.MLG.0000201986.82035.6F. 16585858

[14] C. Hopkins , S. Gillett , R. Slack , V. J. Lund , and J. P. Brown , “Psycometric Validity of the 22‐ Item Sinonasal Outcometest,” Clinical Otolaryngology 34 (2009): 447–454, 10.1111/j1749-4487.2009.01995.x. 19793277

[15] K. J. Rennie , J. O'Hara , N. Rousseau , et al., “Nasal Airway Obstruction Study (NAIROS): A Phase III, Open‐Label, Mixed‐Methods, Multicentre Randomised Controlled Trial of Septoplasty Versus Medical Management of a Septal Deviation With Nasal Obstruction,” Trials 21, no. 1 (2020): 179, 10.1186/s13063-020-4081-1. PMC7020359 32054508

[16] J. Brunsworth , J. Holmes , and R. Sindwani , “Inferior Turbinate Hypertrophy: Review and Graduated Approach to Surgical Management,” American Journal of Rhinology & Allergy 27 (2013): 411–415, 10.2500/ajra.2013.27.3912. 24119606

[17] J. F. Piccirillo , M. G. Merritt , and M. L. Richards , “Psychometric and Clinimetric Validity of the 20‐Item Sino‐Nasal Outcome Test (SNOT‐20),” Otolaryngology—Head and Neck Surgery 126 (2002): 41–47, 10.1067/mhn.2002.121022. 11821764

[18] J. R. Buckland , S. Thomas , and P. G. Harries , “Can the Sino‐Nasal Outcome Test (SNOT‐22) be Used as a Reliable Outcome Measure for Successful Septal Surgery?,” Clinical Otolaryngology and Allied Sciences 28, no. 1 (2003): 43–47, 10.1046/j.1365-2273.2003.00663.x. 12580880

[19] A. L. Poirrier , S. Ahluwalia , A. Goodson , M. Ellis , M. Bentley , and P. Andrews , “Is the Sino‐Nasal Outcome Test‐22 a Suitable Evaluation for Septorhinoplasty?,” Laryngoscope 122, no. 9 (2012): 1983–1987, 10.1002/lary.23615. 22991249

[20] P. Sahlstrand‐Johnson , B. Ohlsson , C. Von Buchwald , M. Jannert , and M. Ahlner‐Elmqvist , “A Multi‐Centre Study on Quality of Life and Absenteeism in Patients With CRS Referred for Endoscopic Surgery,” Rhinology 49 (2011): 420–428, 10.4193/Rhino11.101. 21991567

[21] P. Sahlstrand‐Johnson , C. Hopkins , B. Ohlsson , and M. Ahlner‐Elmqvist , “The Effect of Endoscopic Sinus Surgery on Quality of Life and Absenteeism in Patients With Chronic Rhinosinuitis: A Multi‐Centre Study,” Rhinology 55, no. 3 (2017): 251–261, 10.4193/Rhino16.126. 28624844

[22] P. M. Fayers and D. Machin , Quality of Life: The Assessment, Analysis, and Interpretation of Patient‐Reported Outcomes (John Wiley & Sons, 2007), 10.1002/9780470024522.

[23] S. Carrie , T. Foutweather , T. Homer , et al., “Effectiveness of Septoplasty Compared to Medical Management in Adults With Obstruction Associated With a Deviated Nasal Septum: The NAIROS RCT,” Health Technology Assessment 28, no. 10 (2024): 1–213, 10.3310/MVFR4028. PMC11017631 38477237

[24] T. Harju and J. Numminen , “Factors Predictive of Outcome in Inferior Turbinate Surgery,” Ear, Nose, & Throat Journal 101, no. 7 (2022): 443–448, 10.1177/0145561320966066. 33064011

[25] K. Sleurs , J. Postelmans , and J. Smit , “Radiofrequency Ablation for Inferior Turbinate Hypertrophy: Predictive Factors for Short and Long‐Term Outcomes,” Annals of Otology, Rhinology, and Laryngology 132, no. 8 (2023): 888–894, 10.1177/00034894221121407. 36082420

[26] A. Weaver and A. Wood , “Promoting Equity When Using the SNOT‐22 Score: A Scoping Review and Literature Review,” Sinusitis 6, no. 1 (2022): 15–20, 10.3390/sinusitis6010002.

[27] S. Sinno , K. Mehta , Z. H. Lee , S. Kidwai , P. B. Saadeh , and M. R. Lee , “Inferior Turbinate Hypertrophy in Rhinoplasty: Systematic Review of Surgical Techniques,” Plastic and Reconstructive Surgery 138, no. 3 (2016): 419e–429e, 10.1097/PRS.0000000000002433. 27556616

[28] D. Passali , M. Loglisci , L. Politi , G. C. Passali , and E. Kern , “Managing Turbinate Hypertrophy: Coblation vs. Radiofrequency Treatment,” European Archives of Oto‐Rhino‐Laryngology 273, no. 6 (2016): 1449–1453, 10.1007/s00405-015-3759-6. 26321749

[29] A. A. Mirza , T. A. Alandejani , H. Y. Shawli , M. S. Alsamel , M. O. Albakrei , and H. M. Abdulazeem , “Outcomes of Microdebrider‐Assisted Versus Radiofrequency‐Assisted Inferior Turbinate Reduction Surgery: A Systematic Review and Meta‐Analysis of Interventional Randomised Studies,” Rhinology 58, no. 6 (2020): 530–537, 10.4193/Rhin19.350. 32679584

