# Supplementary material for: Nasal Obstruction Is Reduced 5–7 Years After Radiofrequency Turbinate Reduction: Results From a Retrospective Observational Cohort Study
Source: Clin Otolaryngol. 2026 Jul 24;51(6):914–22. doi: 10.1111/coa.70148 (PMC13631798; doi:10.1111/coa.70148)
Supplement: Supplementary file 1 [file COA-51-914-s001.docx]

**Suppl. 1.** Patients scoring severe conditions in the sleep function domain, showing significant improvement for all criteria (p<0.05).
